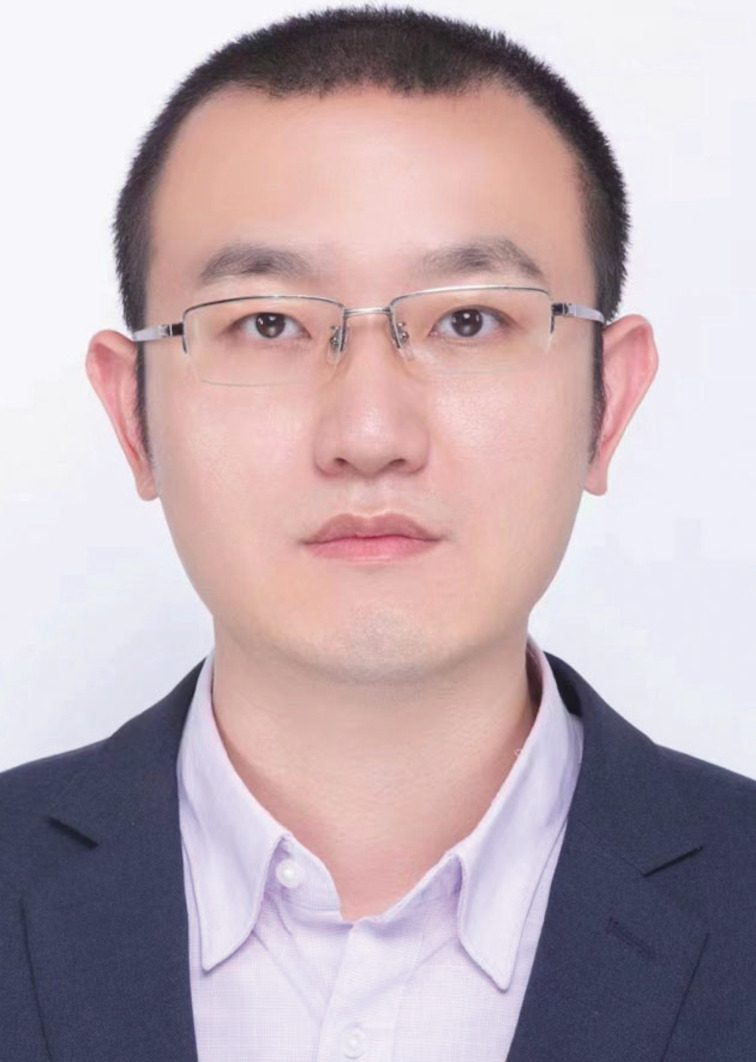# Chemical and Biological Strategies for Profiling Protein‐Protein Interactions in Living Cells

**DOI:** 10.1002/asia.202300226

**Published:** 2023-05-05

**Authors:** You‐Yu Wang, Wenyi Li, Bang‐Ce Ye, Xiao‐Bao Bi

**Affiliations:** ^1^ Collaborative Innovation Center of Yangtze River Delta Region Green Pharmaceuticals & College of Pharmaceutical Sciences Zhejiang University of Technology Hangzhou 310014, Zhejiang Province P. R. China; ^2^ Department of Biochemistry and Chemistry, La Trobe Institute for Molecular Science La Trobe University Victoria 3086 Australia

**Keywords:** protein-protein interactions, living cells, fluorescence, proximity labeling, enzyme, photoactivation

## Abstract

Protein‐protein interactions (PPIs) play critical roles in almost all cellular signal transduction events. Characterization of PPIs without interfering with the functions of intact cells is very important for basic biology study and drug developments. However, the ability to profile PPIs especially those weak/transient interactions in their native states remains quite challenging. To this end, many endeavors are being made in developing new methods with high efficiency and strong operability. By coupling with advanced fluorescent microscopy and mass spectroscopy techniques, these strategies not only allow us to visualize the subcellular locations and monitor the functions of protein of interest (POI) in real time, but also enable the profiling and identification of potential unknown interacting partners in high‐throughput manner, which greatly facilitates the elucidation of molecular mechanisms underlying numerous pathophysiological processes. In this review, we will summarize the typical methods for PPIs identification in living cells and their principles, advantages and limitations will also be discussed in detail.

## Graphical abstract

**The ability** to explicate the complicated protein‐protein interactions (PPIs) network can greatly advance the understanding of the mechanisms underlying virtually all the cellular events. This review summarizes the chemical and biological strategies developed to monitor, analyze and identify the PPIs in living cells with highlights on their limitations and challenging questions that need to be addressed.

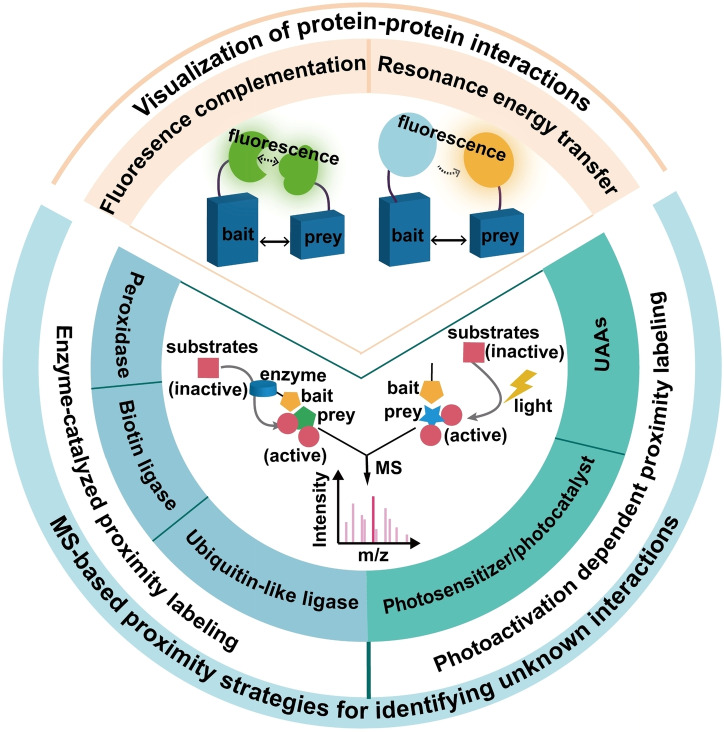

## 1 Introduction

PPIs are essential for intracellular signal transduction and transcriptional regulation, taking integral roles in a wide range of biological processes.^[1]^ Disruption of these PPIs in the intricate interaction networks may lead to altered cellular behaviors and the pathogenesis of various diseases. Thus, the exploration of complex networks could provide informative clues for deciphering the mechanisms underlying the disease progression.^[2]^

In recent years, several PPIs have emerged as validated drug targets for tumor immunotherapy. For instance, the interaction between programmed cell death protein (PD‐1) and its ligand programmed death‐ligand (PD‐L1) could assist the immune surveillance escape, thereby promoting cancer progression.^[3]^ Targeting PD‐1/PD‐L1 pathway using the inhibitors such as nivolumab^[4]^ and cemiplimab^[5]^ have been shown to be beneficial for some patients by activating the immune system. Therefore, elucidating the functions of PPIs and developing inhibitors that can selectively and precisely inhibit one PPI of target will be of great significance for the targeted interventions to control and treat diseases. However, the complex and intersecting protein interaction networks greatly increase the difficulty to analyze the interactions with spatiotemporal resolution in living cells and *in vivo* animals.

Traditional methods such as co‐immunoprecipitation^[6]^ or pull down^[7]^ allow for the identification of numerous potential interaction candidates from crude lysates in a short period of time and thus are regarded as relatively high‐throughput and useful techniques to understand protein interactions. However, these approaches suffer from several intrinsic drawbacks, such as the disruption of all native subcellular microenvironments, the loss of potentially important transient or weak interactions during sample preparation, or the unexpected non‐specific labeling. Comparably, the advantage of yeast two‐hybrid system lies in the ability to characterize PPIs in physiological conditions achieved by utilizing the reconstitution of an active transcription factor,^[8]^ which thus opens the door to exploration of protein interactions in living cells. However, this technique can only suggest the potential interaction of two POIs rather than the interaction that actually occurs in the native cellular context and is also subjected to negative interference and low efficiency of false positive conversion.^[9]^ To address these limitations, many robust techniques have been developed in the past decades, such as the PROPER‐seq that can reveal and map PPIs at the transcriptome scale by high‐throughput sequencing,^[10]^ offering excellent opportunities to elucidate the functions of proteins and the underlying mechanisms of cellular events associated with PPIs. Therefore, as one of the research hotspots in the field of chemical biology, it is necessary to provide an updated summary of the emerging methodologies and applications for profiling PPIs in different biological systems. Herein we provide an overview of chemical and biological approaches currently used for PPIs research, especially with a focus on those applicable to living cells/animals. We further describe how those approaches can be used to address important questions that are not available by traditional methods, hopefully shedding new light on the future direction of methodology development and novel biomedical application explorations.

## 2 Fluorescence and luminescence‐based approaches

Fluorescence and luminescence‐based methods have been widely used to track and analyze protein interactions within cells. Superior to the traditional methods like co‐immunoprecipitation or pull‐down assay, intracellular imaging by combining fluorescent molecules with microscopy techniques can allow real‐time monitoring of interaction strength in nearly natural conditions. In addition, the simultaneous visualization and detection of multiple interactions can provide huge potential to detect drug‐related multiple interactions and explicate the cellular functions of complex interaction networks associated with multiple proteins.^[11]^

### 2.1 Fluorescence fragments complementation assay

**Figure 1:**
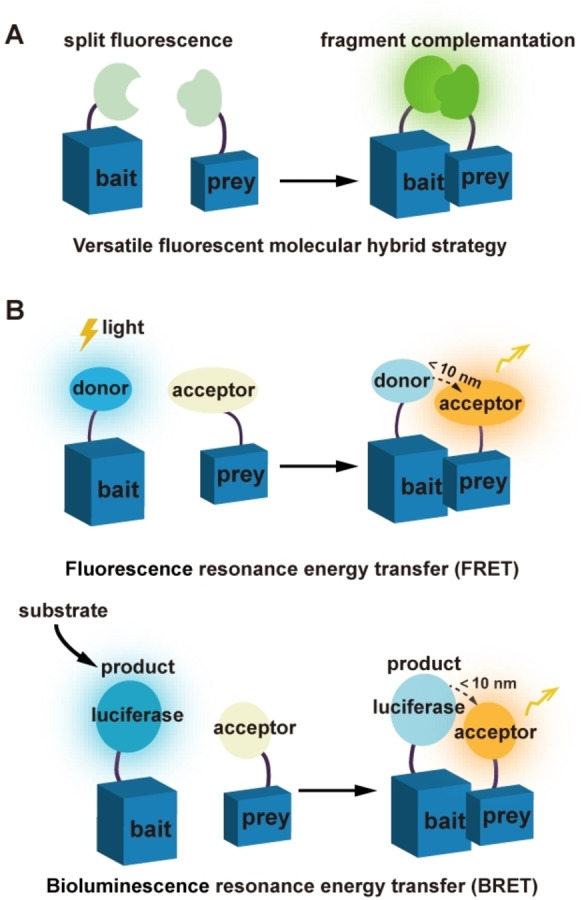
Schematic representation of the principle of fluorescence and luminescence‐based approaches. (A) The two split non‐fluorescent fragments can assemble again once the bait and prey protein interact with each other, finally yielding the functional fluorescent protein to emit fluorescence signal. (B) In FRET and BRET, the interaction between bait and prey protein will make the attached donor and acceptor close to each other, and then the energy transfer occurs, resulting in fluorescence emission at a specific wavelength. Both FRET and BRET rely on energy sources for the energy transfer.

This method relies on the ability of the split non‐functional fragments to assemble at relatively close range when the loaded proteins interact together, yielding the functional fluorescent molecules that can emit fluorescent signals, which is indicative of occurrence of interactions (Figure 1A). The advantage to this technique is that it can be carried out in a physiological environment to characterize the interactions with spatiotemporal resolution.^[12]^ Among these, bimolecular fluorescence complementation (BiFC) is a commonly used visualization tool to determine whether the target POI interacts with a partner and the localization of the interaction occurs within the cell, thus may provide insight into the molecular mechanism involved in diseases progression or for drug evaluation. For example, Parkinson's disease (PD) and Alzheimer's disease (AD) are neurological disorders that are both characterized by pathological accumulation and aggregation of different amyloidogenic proteins, such as α‐synuclein (αSyn)^[13]^ and amyloid‐β (Aβ),^[14]^ respectively. But the exact molecular mechanism remains elusive, and no effective therapeutic drug is available so far. The BiFC assay offers such an opportunity to study αSyn‐Tau interactions in different rodent models *in vivo*, thereby greatly facilitating the screening of potential drugs to boost or inhibit this interaction for therapeutic intervention.[^15^, ^16^]

The BiFC technology mainly uses of two different types of fluorescent molecules: fluorescent proteins such as YFP^[17]^ or phytochromes.^[18]^ Compared to fluorescent proteins‐based system, phytochromes can remain much more stable under physiological conditions and produce a longer fluorescence wavelength, which make them applicable in diverse experimental settings.^[19]^ For example, a tandem fluorescence complementation system based on a split near‐infrared phytochrome, IFP2.0, could exhibit similar photophysical properties but twice intensity as the single‐copy parent protein when was used to image PPIs *in vivo*. By using this tool, the interaction between HIV‐1 integrase (IN) and cellular cofactor protein Lens epithelium‐derived growth factor (LEDGF/p75) was analyzed in live cells, providing some new information to design drug candidates for anti‐HIV therapies.^[20]^ In addition, the smaller signal module is also advantageous to reduce the potential adverse effect on proteins, which may allow to closely recapitulate a network of interactions in the natural state. For instance, a single‐domain of cyanobacteriochrome named as miRFP670nano with a molecular weight of 17 kDa,^[21]^ which is smaller than GFP and bacterial phytochromes, has been applied in BiFC and trimolecular fluorescence complementation (TriFC) sensing system to identify and visualize PPIs in living cells with higher photostability and cellular stability compared to other systems.^[22]^

In addition, combined use of BiFC with other techniques can allow to study much more complicated PPIs. For example, multiple PPIs can be imaged simultaneously by combining large‐Stokes‐shift fluorescent proteins based BiFC assays with donor mT‐Sapphire and acceptor CyOFP1,^[11]^ which provides an important tool to decipher characteristics of signaling pathways. Similarly, the BiFC technology was also combinedly used with two resonance energy transfer techniques simultaneously to identify interacting G‐protein coupled receptors (GPCRs) and quantify interactions between GPCRs and the interacting ancillary proteins, including G proteins and other allosteric modulators of the function of G protein complex.^[23]^ This technology achieved the detection and visualization of up to four distinct proteins simultaneously in the same cell and provided a significant addition to the toolbox for the study of PPIs involving multiple proteins.

However, it should be noted that the fluorescent molecular fragments may also spontaneously assemble in the intracellular environment,^[24]^ especially at a high expression level, which potentially leads to enhanced false‐positive signal values. To this end, one system named Background Assessable and Correctable‐BiFC (BAC‐BiFC) was successfully developed, in which two split fluorescent proteins fused with interacting proteins served as a reference maker and another single fluorescence ratio of initial BiFC signal to the reference signal was used as appropriate condition reference.^[25]^ In addition, the temporal resolution of BiFC analysis is limited by the time required for chromophore formation as the maturation of eventually stable BiFC complex is slow (T_1/2_∼1 h),^[26]^ as well as the stabilization of complexes by the irreversible association of the fluorescent protein fragments *in vivo* also renders it not applicable to interpret dynamic changes of PPIs. Despite these shortcomings, BiFC still remains a sensitive and enabling approach for visualization of subcellular localization of protein complex in living cells.

### 2.2 Resonance energy transfer techniques

Energy transfer plays an important role in the interaction exploration, and its efficiency depends on spectral overlap, distance and relative orientation between donor and recipient molecules. When two POI interact with each other, the fused fluorophores will undergo energy transfer and give off a fluorescent signal within 10 nm, which can be leveraged to detect and characterize PPIs.^[27]^ Particularly, compared to BiFC system, no exact contact is needed between the two molecules, thereby this technique may reduce adverse effect on the actual interactions caused by steric hindrance.

#### 2.2.1 Fluorescence resonance energy transfer (FRET) strategy

FRET depends on the non‐radiative energy transfer between two molecules in close proximity, and the energy transfer efficiency is strongly dependent on the distance. Once a pair of interacting POI is coupled with two chromophores respectively, the acceptor chromophore will emit fluorescence in response to the energy transfered from the activated donor at the time when the two fluorophores are close enough, by which the interaction can be monitored and analyzed (Figure 1B). For example, FRET was applied to analyze the strength variation of interaction between GroEL and GroES in a bacterial protein folding system, showing its potential capability to study the thermodynamics or kinetics of a protein interaction event even though the structural information of proteins is not available yet.^[28]^ Moreover, it was found that one 10‐fold increase in the interaction affinity could result in 0.05 unit increase in FRET efficiency, by which the dissociation constant over a range of mM to nM can be measured, providing the intuitive data to illustrate the interaction in detail.^[29]^ In addition, coupled with fluorescence lifetime imaging microscopy (FLIM)^[30]^ or flow cytometry,[^31^, ^32^] the information about PPIs in live cells can also be acquired quantitatively. In this sense, the combined use of different techniques will allow the design and development of more robust strategies to explore PPIs from different perspectives.

Single‐molecule FRET (smFRET) is another widely used technique in cell biology research to detect the behavior of individual molecules in real‐time with functional cycle, as it can overcome the limited spatial resolution of traditional FRET techniques.^[33]^ For example, smFRET has been applied to elucidate the functional distinctions between two periplasmic chaperones, Skp and SurA, involving in the biogenesis of *E. coli* β‐barrel outer membrane proteins.^[34]^ However, this method is limited by irreversible photobleaching, and not applicable for long‐term observation of living cells due to the cytotoxic photodamage. To this end, DyeCycling strategy was developed as a novel measurement tool to conquer the photobleaching limitation by reversibly controlling the binding and separation of dyes to the biomolecular of interest. The reversible dye binding scheme such as site‐specific reversible binding mediated by canonical amino acids (e. g., cysteines) enables reposted dye replacement, by which the observation time is no longer limited by photobleaching of only a single dye.^[35]^ In addition, another photoswitching FRET (psFRET) technology requires less illumination light and can be reactivated again to repeat the experiment, which thus allows for the monitoring of dynamics and/or averaging of responses to improve signal to noise ratio.^[36]^ With the utility of psFRET as a readout, the activity of a key regulator of the apoptotic response, caspase‐3, could be successfully monitored, showing the heterogeneous cellular responses to staurosporine treatment.^[36]^ Therefore, these advanced techniques make FRET being an indispensable tool to study PPIs.

#### 2.2.2 Bioluminescence resonance energy transfer (BRET) strategy

Over the last decades, BRET has been extensively used for monitoring PPIs in living cells, particularly for the interactions associated with GPCR.^[37]^ In BRET strategy, the luminescence system involves a luciferase and a fluorescent protein (FP), notably both of which can emit fluorescence. Once the FP receives the bioluminescence initiated by an enzymatic reaction in the presence of the luciferase and substrate, it will then emit the fluorescence. Thus, the detected signal indicates that the coupled POI can interact with each other.^[38]^ Compared to FRET, BRET strategy not only integrates the attractive advantages of FRET, but also avoids the tricky issues such as phototoxicity and photobleaching caused by excitation illumination.^[39]^

So far the most commonly used luciferases in BRET are the sea pansy derived Renilla luciferase (Rluc) and its mutated derivative Rluc8. The first direct experimental evidence for CK2 holoenzyme aggregates in the cell was revealed by utilizing a BRET pair consisting of the fusion proteins CK2α‐Rluc8 and CK2α‐GFP^2^.^[40]^ In addition to this, one smaller luciferase subunit Nanoluciferase (Nluc) (19 kDa) is also available, which was developed from a large multi‐component luciferase isolated from the deep sea shrimp *Oplophorus gracilirostris*.^[41]^ Excitingly, it produces an intense and sustained luminescence signal that allows the accurate quantification of very small numbers of entities. For example, Nluc has been used to develop the caspase sensor that can display a robust 10‐fold decrease in BRET ratio upon the joint linker is cleavage by caspase, providing a suitable tool to measure the intracellular caspase activity with modest specificity.^[42]^ Besides, to date various acceptor molecules and substrates such as Venus or furimazine are already available.^[43]^ This further provides us a good opportunity to select and use suitable BRET pairs for our intended application studies. Overall, in terms of the sensitivity, robustness, and suitability for real‐time measurements with a high responsiveness, BRET‐based techniques are still attractive tools to study PPIs in future.

## 3 Mass spectrometry (MS) based proximity labeling strategies for profiling novel PPIs

Although the aforementioned fluorescence and luminescence‐based approaches are particularly suitable for real‐time analysis of known PPIs in living cells, they are also suffering from the inability to explore unknown PPIs. Therefore, many MS‐based proximity labeling strategies have been developed and provide such excellent solutions to this problem, thus enabling the analysis and identification of the large portion of unknown PPIs including the weak/transient ones even without any prior information. The ability to rapidly analyze complicated samples after digesting out of cross‐linking mixtures has made MS as an indispensable tool to efficiently identify unknown biomolecules in life science and chemical biology.[^44^, ^45^] Furthermore, in combination of proximity‐based capturing strategies, MS has been widely used to identify the interacting proteins from the large pull‐down protein mixtures, emerging as the reliable tool to address the challenging biomedical questions that cannot be addressed by previous traditional techniques. For example, the dynamic organization of sequential events in autophagy that is highly regulated by PPIs has been uncovered with the right spatial and temporal resolution by using MS technology.^[46]^ In addition, several robust proximity‐based chemical crosslinking strategies make it possible to study the weak and transient PPIs in cells by converting these dynamic and noncovalent interactions into stable covalently‐linked protein complex. This section will highlight the principles, limitations, and progress of these emerging tools.

### 3.1 Enzyme‐catalyzed proximity labeling

Enzymes are very attractive tools in various disciplines due to their featuring characteristics such as mild conditions, biocompatibility, and high substrate specificity. To date, several enzymes have been developed as robust tools to label the nearby proteins in defined microenvironment, including peroxidase, biotin ligase, ubiquitin‐like ligase and among others.^[47]^ This strategy is mainly dependent on the ability of enzymes to convert their substrates into chemically active substances or intermediates, which then diffuse out of the active site in a limited radius range to react with nearby proteins for tagging. Subsequently, the labeled proteins are harvested by coated beads and identified by MS‐based proteomics technology.^[48]^

#### 3.1.1 Peroxidase

**Figure 2:**
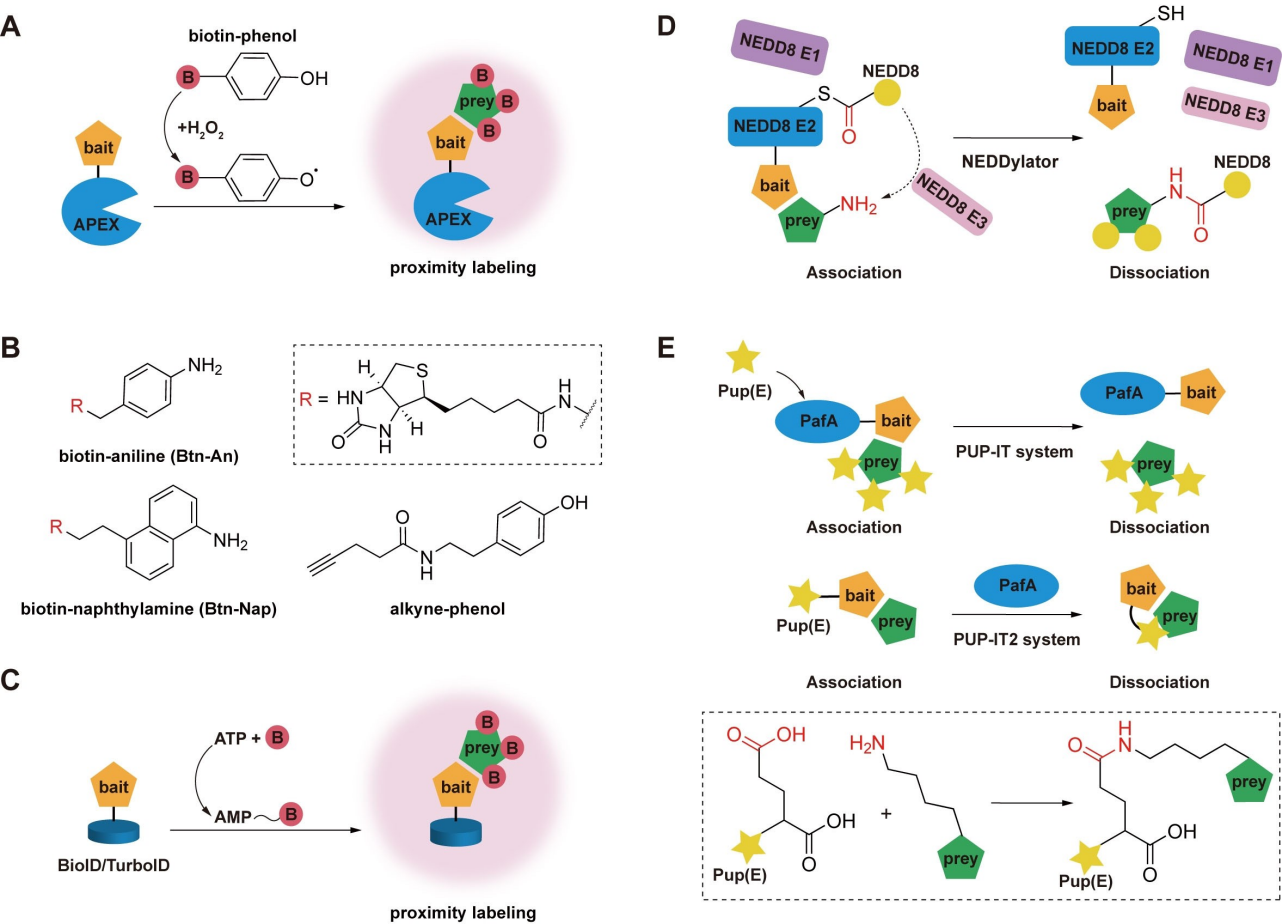
Schematic illustration of enzyme‐catalyzed proximity strategies for labeling proximal proteins. (A) Proximity labeling triggered by APEX enzyme. In the presence of H_2_O_2_, APEX converts the substrates into active free radicals that can be subsequently trapped by the adjacent residues, resulting in the labeling of prey protein in close proximity (Adapted with permission from reference [55]. Copyright 2014, Nature Publishing Group, a division of Macmillan Publishers Limited. All Rights Reserved). (B) Examples of chemical structures of APEX2 substrates. (C) Proximity labeling catalyzed by biotin ligases‐derived enzymes. BioID/TurboID uses ATP and biotin to generate biotin‐5′‐AMP, which is a reactive intermediate that can be attached to the prey protein via covalent bond formation, resulting in biotinylation. (D) NEDD8 conjugation machinery‐mediated proximity labeling. With the action of E1, E2, E3, NEDD8 is linked to E2 via a labile thioester bond at the catalytic center and then can be transferred and conjugated with proximal lysine residues on the prey and/or bait proteins. (E) In PUP‐IT (up panel) and PUP‐IT2 system (down panel), free Pup(E) or Pup(E) fused with a bait protein can be activated by PafA and linked to it via the labile thioester bond, and then transferred and conjugated to nearby proteins close to the bait via the isopeptide bond formation shown in the dotted rectangle (Adapted with permission from reference [87]. Copyright 2018, Qiang Liu et al and from reference [91]. Copyright 2022 Yue, Xu, Cao and Zhuang, respectively).

Peroxidase‐mediated proximity labeling offers fast reaction kinetics by using free radical chemistry^[47]^ (Figure 2A). The first peroxidase used to study protein interactions was horseradish peroxidase (HRP). It is widely used as antibody conjugates for detecting proteins of interest in the lysates of biological samples by generating chemiluminescent signals for Western blots and chromogenic signals for ELISAs.^[49]^ However, the active HRP contains four structurally‐essential disulfide bonds that are susceptible to intracellular reducing environment,^[50]^ leading to the application restricted to the oxidizing secretory pathway and cell surface.^[51]^

Unlike the HRP, the APEX, derived from dimeric pea or soybean ascorbate peroxidases, is a monomeric peroxidase reporter which lacks disulfides and calcium binding sites and hence can be expressed in the reducing cytosolic environment of cells without loss of activity.^[52]^ For example, by knocking in APEX gene to the endogenous locus of lysosome‐associated membrane protein 1 (LAMP1), the proteomic microenvironment of the neuronal endolysosomal network could be captured and characterized, by which new lysosomal membrane and lysosomal‐interacting proteins in human iPSC‐derived neurons can be identified.^[53]^ However, one critical limitation associated with APEX lies in its low sensitivity. Although certain compounds such as disuccinimidyl suberate (DSS) could be added to improve the capturing efficiency and accuracy through chemically crosslinking labeled proximal proteins,^[54]^ the low efficiency in the labeling process is irretrievable. To this end, a much more active APEX2 was subsequently developed by using yeast display‐based evolution strategy.^[55]^ With the high catalytic efficiency, APEX2 has been validated to be suitable tool for elucidating microprotein‐protein interactions (MIPs) and identification of glycan‐binding proteins.^[56]^ Also, due to the rapid labeling ability, APEX2 is especially applicable to profile and capture functional proteins in special cellular structures, such as insoluble nuclear lamina.^[57]^ In recent years, APEX2 has also been utilized to characterize the proteome of subcellular compartments and transient aggregates.^[58]^ In addition, to selectively target numerous cellular compartments and structures, split APEX has also been developed to control the enzyme function in highly specific subcellular regions that are inaccessible by single‐gene constructs.[^59^, ^60^]

To date, phenoxyl radicals generated by one‐electron oxidation of phenol have been widely used for APEX‐mediated labeling due to the high reactivity and short lifetime, which can avoid non‐specific aggregation.^[61]^ To further improve the labeling efficiency and accuracy, a series of newly expanded substrates have also been designed and synthesized by incorporating some chemical modifications into the aromatic rings to endue them with improved properties[^62^, ^63^] (Figure 2B). For example, it was found that alkyne‐phenol not only had enhanced cell membrane permeability, but also the labeled proximal protein with an alkyne handle can be further derivatized with biotin‐conjugated azide using click chemistry reaction for visualization and enrichment.^[64]^ Despite these advances, the greatest potential drawback of peroxidases is the inevitable cell damage and the disturbance to redox‐sensitive pathways caused by hydrogen peroxide.^[65]^ Although free radical quenchers, including sodium ascorbate and trolox, and peroxidase inhibitor such as sodium azide are commonly used to terminate the labeling reaction to avoid excessive oxidation,^[64]^ cautions need to be taken that these additives can still cause cell damage in certain ways.^[66]^

#### 3.1.2 Biotin ligase

BirA is a member of a family of enzymes called biotin protein ligases, which are responsible for post‐translationally attaching biotin to a specific lysine ϵ‐amino group within a defined short peptide substrate in the presence of adenosine triphosphate (ATP).^[67]^ Interestingly, while a single R118G mutation located in the biotin‐binding motif can cause the enzyme to lose its original substrate stringency, this mutant still can randomly biotinylate any protein with ϵ‐amino groups of lysines within its proximity,^[68]^ thus making it quite useful for labeling proximal proteins (Figure 2C).^[69]^ For example, BioID has been applied to study the microtubule plus‐end tracking protein SLAIN2, and to generate its proximity interaction map through *in vivo* proximity‐dependent biotin identification, which can provide an important resource for future elucidating SLAIN2 functions at the centrosome.^[70]^ Moreover, compare with BioID, BioID2 can allow more‐selective targeting of fusion proteins, require less biotin supplementation, and exhibit enhanced labeling of proximal proteins. Fusing BioID2 to the endogenous structural membrane protein Junctophilin 2 coding sequence can help to investigate the cardiac dyad proteome in living cardiomyocytes, which eliminated the use of tedious procedures involving extracting hydrophobic membrane proteins.^[71]^ Since the fusion of POI with relatively large labeling tags (BioID, 35 kDa) sometimes results in improper biophysical functions owing to undesired interactions with other molecules, alternative strategies have been successfully developed. One of such popular examples is through the combined use of FKBP‐FRB dimerization system (FK506‐binding protein: FKBP‐rapamycin‐binding domain of mammalian target of rapamycin [mTOR]) with BioID. The merit of this method is that it can draw BioID, which was fused with FKBP, close to the POI carrying FRB for proximity labeling only after the rapamycin analogue AP21967 was added into the cell culture to induce FKBP‐FRB dimerization.^[72]^ Therefore, as FRB is a much smaller protein only with a molecular weight of 11 kDa,^[73]^ it may have less adverse effect on the normal functions of POI before performing the biotin labeling. Recently, AirID has been designed as a novel proximity biotinylating enzyme by using an ancestral enzyme reconstruction algorithm and metagenome data based on BirA. Compared to BirA, AirID has both high biotinylating activity and proximity dependency.^[74]^ Also, it has been proved that AirID‐fusion proteins are able to accurately map each well‐known interactor in the transient‐ and stable‐expression cells and can be used in animal models and plants.^[75]^ For instance, one platform based on proximity‐dependent biotinylation by AirID has been developed to identify drug‐induced neo‐substrates of the E3 ligase cereblon (CRBN), demonstrating the potential to analyze various protein degraders or understand the drug‐inducible interactomes.^[76]^

Compared with peroxidase, BioID‐based methods have several appealing features, such as no toxic reagents are needed, use of highly soluble biotin as substrate and intracellular ATP to initiate the reaction. However, the conventional labeling mediated by BioID requires 15–18 h which may increase the non‐specific background values. Thus, TurboID (35 kDa) and miniTurbo (28 kDa) obtained through directed evolution of BioID have been developed to allow efficient proximity labeling within 10‐mins.^[77]^ Recently, TurboID is used to identify high‐confidence interactors of the Lassa virus polymerase, shedding light on the potential druggable target for host‐directed antivirals development.^[78]^ However, possibly due to the higher activity of TurboID than that of miniTurbo, low‐level labeling often occurred even without addition of exogenous biotin into cells, indicating that endogenous biotin can still serve as the substrate for labeling. In this sense, TurboID is not a suitable tool for the study that needs to be carried out in a precisely defined labeling time window.^[77]^

To better control of the enzyme activity, the split strategy has been adopted to develop split‐BioID^[79]^ and split‐TurboID,^[80]^ which will be reconstituted after ligation driven by a pair of specific contact. Additionally, a set of split TurboID variants have shown the ability to reversibly assemble in the presence of certain light,^[81]^ which may offer a convenient tool to control the enzyme activity triggered by light. As these split enzymes can only reassemble and function at a certain distance, these split fragmentation strategies are particularly suitable for studying intracellular regions with less incidence of false positives, such as organelle contact sites. Importantly, these enzymes can be easily expressed with POI within live cells and several animal models,^[82]^ which may allow to accurately profile the interacting proteins in native states. Recently, they have been used to map the SARS‐CoV‐2 virus‐host interactome in human cells to reveal underlying mechanisms of pathogenesis.[^83^, ^84^]

#### 3.1.3 Ubiquitin‐like ligase

In addition to the above tools, another category of useful enzyme‐based proximity techniques includes NEDDylator and PUP‐IT (Pupylation‐based interaction tagging), which are derived from the ubiquitination‐like machinery that mediate the post translational modifications of protein substrates with NEDD8 or Pup(E) in living cells. NEDDylator is a powerful proximity‐catalytic tagging tool, which relies on the fusion of a NEDD8 E2‐conjugating enzyme to the bait protein and permits transfer of the ubiquitin homolog NEDD8 to the prey proteins with the action of NEDD8 E1‐activating enzyme and NEDD8 E3 ligases^[85]^ (Figure 2D). The system has been verified to identify substrates for specific E3 ligases which considered to be challenging as the interactions are weak and ubiquitinated proteins are subjected to rapid proteasomal degradation.^[85]^ This unique covalent tagging tool can also be used to identify the small molecule binding proteins such as the drug dasatinib and its endogenous protein binders^[86]^ which further unleash the great potential of NEDDylator in identifying protein‐protein interactomes.

Similar to NEDDylator, PUP‐IT system is innovatively developed from the pupylation machinery existing in the several bacteria species, by which prokaryotic ubiquitin‐like protein (Pup) can be covalently attached to the side chain of lysine residue on the target proteins as the form of Pup(E) catalyzed by the Pup ligase PafA in the presence of ATP for subsequent degradation. Interestingly, the conjugation mediated by PafA does not require any substrate specificity, thus when PafA is fused to a POI, it will ligate Pup(E) to nearby lysine residues on the fused protein (bait), as well as any proteins (prey) that are interacting with the bait without the requirement of specific sequences (Figure 2E). To date PUP‐IT has been successfully to identify the interacting partners of a critical costimulatory receptor CD28 for T lymphocyte activation^[87]^ and explore the roles of one mitochondria‐associated ubiquitin ligase MARCH5 on peroxisomes.^[88]^ As all the components of PUP‐IT system can be expressed in cells, it has the great potential to be used in living animals.^[87]^ However, because Pup is a larger substrate compared with the biotin‐based small molecules, designing shorter substrates may increase the flexibility and improve the labeling efficiency of this system. It was found that the C‐terminal 26 amino acids of Pup was sufficient for PafA‐mediated labeling, indicating that there is no need to use the full length of Pup as the substrate.^[89]^ For example, DE28, which is a truncated form of Pup (D37 to E64), can still be conjugated to target lysine residues with comparable efficiency to wild type. Moreover, by using yeast surface display system, a shorter peptide substrate composed of only 14 amino acids was also successfully identified.^[90]^ It not only has the advantage of the restriction to form special modification only at a single lysine site, but also can be chemically modified with different tags at the N terminus to allow multiple modification on target proteins to improve the capture efficiency.^[90]^ Despite these attractive features of PUP‐IT system, PafA is a larger enzyme than BirA* or APEX, and fusion of it with a bait protein may cause the adverse effect on the protein activity, thus alternative strategy has been reported recently in which the bait protein was fused with much smaller Pup(E) instead of large PafA^[91]^ and termed as PUP‐IT2 (Figure 2E), offering one alternative tool for proximity labeling in living cells. Altogether, compared with other types of enzymes‐catalyzed proximity labeling techniques, NEDDylator, PUP‐IT, and PUP‐IT2 have lower non‐specific backgrounds due to higher specificity and narrower radius for labeling as the active Pup and NEDD8 intermediate cannot diffuse freely from the vicinity of the enzyme.^[87]^ The basic information and respective limitations of these tools are summarized in Table 1.

**Table 1:** Overview of the characteristics of enzymes used for proximity labeling.

Enzyme		Type	Size (kDa)	Substrates	Modification site	Labeling time	Labeling range	Limitations
HRP		Peroxidase	44	H_2_O_2_, biotin phenol or other	Tyr, Trp, Cys, His	1 min	∼270 nm^[92]^	The toxicity of H_2_O_2_; limited to secretory pathway and cellular membrane applications
APEX		Peroxidase	28	H_2_O_2_, biotin phenol or other	Tyr, Trp, Cys, His	1 min	<20 nm	The toxicity of H_2_O_2_
APEX2		Peroxidase	28	H_2_O_2_, biotin phenol or other	Tyr, Trp, Cys, His	1 min	<20 nm	The toxicity of H_2_O_2_
split APEX2		Peroxidase	/	H_2_O_2_, biotin phenol or other	Tyr, Trp, Cys, His	1 min	<20 nm	The toxicity of H_2_O_2_
BioID		Biotin ligase	35	ATP, biotin	Lys	12–18 h	−10–35 nm	Low catalytic activity
BioID2		Biotin ligase	27	ATP, biotin	Lys	12–18 h	−10–35 nm	Low catalytic activity
split‐BioID2		Biotin ligase	/	ATP, biotin	Lys	12–18 h	−10–35 nm	Low catalytic activity
TurboID		Biotin ligase	35	ATP, biotin	Lys	10 min	−10–35 nm	The possible difficulty to control or minimize background labeling values caused by the high enzymatic activity; potential toxicity in long‐term experiments
miniTurbo		Biotin ligase	28	ATP, biotin	Lys	10 min	−10–35 nm	Lower activity and stability in comparison to TurboID
split‐TurboID		Biotin ligase	/	ATP, biotin	Lys	10 min	−10–35 nm	Lower activity in comparison to split APEX
AirID		Biotin ligase	35	ATP, biotin	Lys	3 h	−10–35 nm	Large size may interfere with interactions especially in cells
NEDD8 E2 ligase Ub12		Ubiquitin‐like ligase	11	NEDD8, NEDD8 E1‐activating enzyme, NEDD8 E3 ligases	Lys	1 h	direct	The requirements for the endogenous NEDD8 pathway
PafA		Ubiquitin‐like ligase	55	Pup(E), DE28, biotin‐peptide 4.1	Lys	30 min	−60–80 A	For PUP‐IT, the large size of PafA may cause the dysfunction of bait protein; For PUP‐IT2, it has lower activity as the POI only fused to one substrate

### 3.2 Photoactivation dependent proximity labeling

The central mechanism in the enzyme‐based strategies just described above is the generation of highly active intermediates or species that can quickly react with certain residues of nearby recipient proteins by the covalent bond formation for subsequent enrichment and MS analysis. Similarly, the reactive free radicals or intermediates can also be generated by using light, thus which provides us alternative toolkit to analyze PPIs with high spatiotemporal resolution in living cells due to the biocompatible conditions and enhanced selectivity.^[93]^

#### 3.2.1 Photosensitizer/photocatalyst mediated labeling

**Figure 3:**
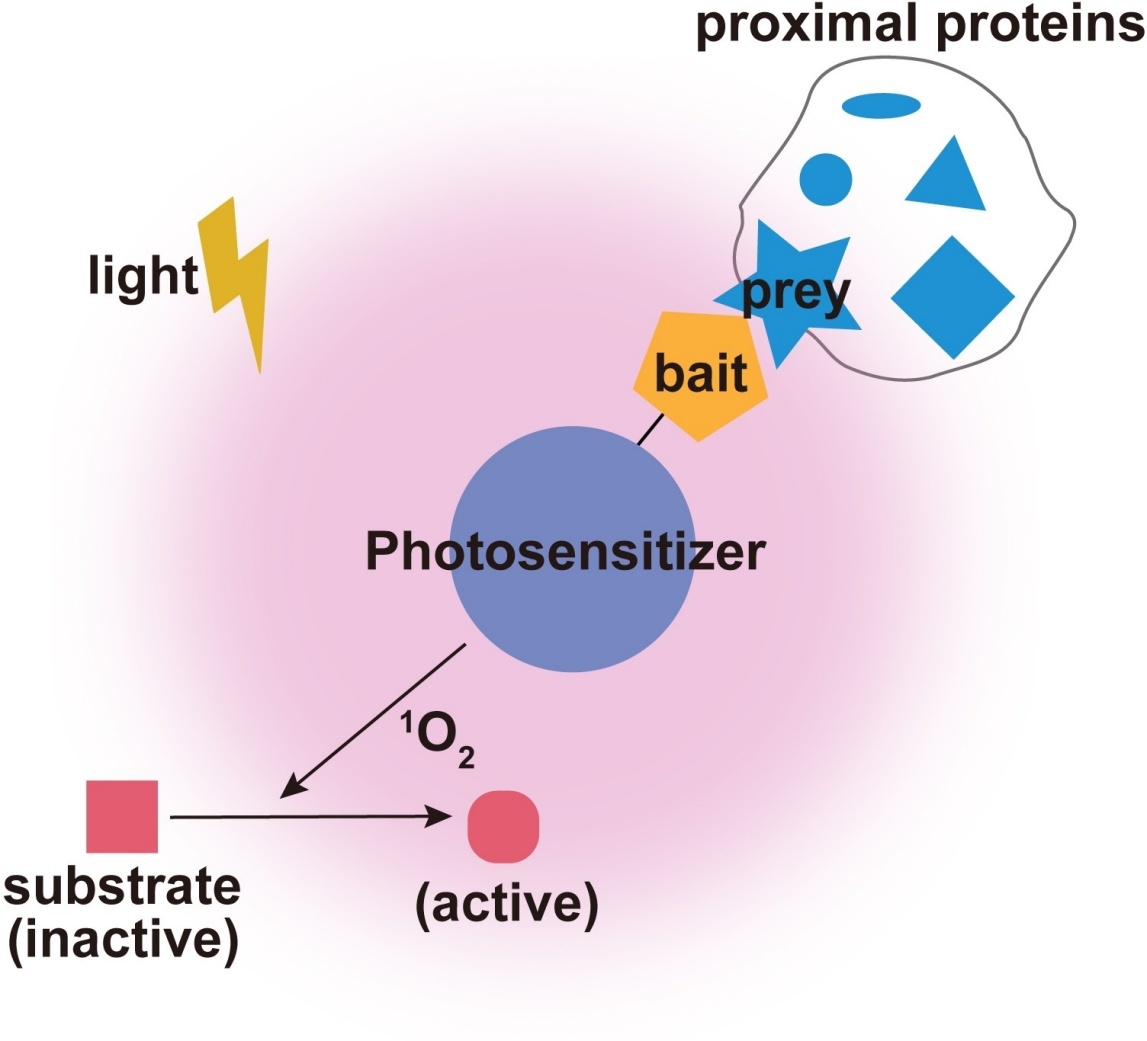
Schematic representation of photosensitizers‐mediated protein complex labeling. Under certain light illumination, the generated singlet oxygen by photosensitizers can convert certain compounds into active species, which can further be conjugated with surrounding proteins in the proximity of POI and photosensitizers. Adapted with permission from reference [99]. Copyright 2022, Springer Nature.

Photosensitizers can generate reactive oxygen species via a complex process of photoreactions.^[94]^ Subsequently, the active singlet oxygen with short lifetime and limited diffusion radius (t_1/2_<0.6 μs in cells)^[95]^ can promiscuously oxidize the neighboring amino acid residues such as tyrosine^[96]^ and histidine,[^97^, ^98^] which leads to umpolung polarity and generates the electrophilic moiety that can further react with amino probe nucleophiles, finally achieving the labeling of interacting proteins.^[99]^ To date, several protein‐based photosensitizers, such as the engineered fluorescent proteins KillerRed or TagRFP, the flavin‐binding proteins miniSOG or Pp2FbFP^L30M^, have attracted wide interests as they are genetically encodable and can be expressed in a fusion protein manner in cells for targeted protein photo‐oxidative activation/desensitization^[100]^ (Figure 3). For example, Skp2, an F‐box protein of the SCF ubiquitin ligase fused with miniSOG, was used as a model and expressed in mammalian cells to capture the nearby proteins in which the biotin‐conjugated thiol molecule could be oxidized and activated by the locally generated singlet oxygen by miniSOG upon blue light illumination to form a disulfide bond with the surface cysteine of its interacting partner Skp1.^[101]^

Except for those large, genetically encoded photosensitizers, small molecule‐based photosensitizers and metal catalyst are alternative powerful toolkits for capturing proximal proteins within the specific organelle of interest. For instance, by utilizing the ability of Hoechst 33342 to effectively bind the DNA minor groove, the photosensitizer dibromofluorescein (DBF) was coupled with Hoechst 33342 to yield DBF‐Hoechst bifunctional molecule as a nucleus‐targeted photosensitizer to identify nuclear proteins with high spatiotemporal resolution.^[102]^ Similarly, DBF can also be used to profile mitochondrial proteome dynamics in living cells by use of the triphenylphosphonium cation as the localizable moiety.^[103]^ In comparison to protein‐based photosensitizers, small molecule photosensitizers have several advantages. For example, firstly they do not require the introduction of exogenous genes into cells for protein expression; Second, they have the great flexibility to be used as a targeted molecules for profiling the proteomes in a specific cellular compartment or microenvironment by utilizing the corresponding targeting moiety.^[104]^

For photocatalyst‐mediated strategies, ruthenium and iridium are commonly used so far which rely on the ability of photocatalyst under certain light illumination to generate highly active species, which can further enable the electrophilic or nucleophilic chemical probes to be conjugated with the proteins in a defined environment. For instance, the ruthenium (II) bipyridine complex (Rubpy) can generate phenoxyl radical and thus be used to label the tyrosine residues of target protein close to the catalyst in a complex protein mixture by using 1‐methyl‐4‐aryl‐urazole (MAUra)‐derived probes as the radical trapping agent.^[105]^ However, the toxicity of ammonium persulfate (APS) used in the photolysis of the ruthenium(II) tris‐bipyridyl dication (Ru(II)bpy_3_^2+^) makes it unsuitable for living cell labeling. To this end, several strategies without the use of APS were subsequently reported. For example, Robin S. Bon and coworkers developed a ruthenium‐bipyridyl (Ru(II)(bpy)_3_) conjugated peptide as the directing ligand for targeting and modifying the protein of target (anti‐apoptotic protein MCL‐1) and found that the MCL‐1 could be successfully labeled even when APS was omitted from the reaction condition.^[106]^ Despite the lower labeling yield, this result indicates the potential of this strategy to study transient/weak PPIs in cells. By leveraging local single‐electron transfer (SEC) mechanism, Hiroyuki Nakamura and coworkers developed [Ru(bpy)_3_]Cl_2_‐conjugated small molecules and found that they can serve as cell‐permeable photocatalytic ligands to selectively target intracellular proteins in intact erythrocytes, thereby revealing its potential to modify native proteins in an intracellular environment in other types of cells.^[107]^ Besides, in the work reported by the same group, a ligand‐conjugated Ru(bpy)_3_ photocatalyst with dual applications was also developed, which can be used not only for target‐selective protein labeling, but also for protein knockdown by chromophore‐assisted light inactivation (CALI) under visible light irradiation in vitro and within cells.^[108]^ Thus, as can be seen from these ongoing efforts made by researchers, couplings of Ru catalyst with different ligands of POI will find wide applications, such as selective modification of intracellular proteins or profiling of transient/weak PPIs in living cells.

For iridium‐mediated proximity labeling, it seems much more popular recently. For instance, through combined use of the pretargeting ability of iridium‐based catalyst and external light, spatiotemporal control of profiling mitochondria proteomics in living cells have been achieved, in which Ir(ppy)_2_bpy was identified as the mitochondria‐targeting catalyst and upon the *para*‐azidobenzyl‐caged phenolic group of the probe was deprotected by the light, this catalyst can *in situ* convert it into highly reactive quinone methide intermediate that further react with the surrounding proteins. This study not only offers a tool to reveal the proteome in the mitochondria with high spatiotemporal precision, but also provides a general photocontrolled proximity labeling strategy for profiling proteomes in some other types of subcellular organelles in future studies.^[109]^ In addition, a powerful catalyst‐driven proximity labeling strategy termed as MicroMap (μ Map) has also been developed, in which iridium catalyst was used to activate diazirines‐based probes to generate active carbene species with blue light by a Dexter energy transfer mechanism. Compared to UV activation, this technique could localize the generation of carbenes to within 0.1 nm of the photocatalyst, thus producing minimal background signal andshowing the great potential for the antibody target identification, investigation of signal transduction pathways and profiling cellular microenvironments.^[110]^ Subsequently, to decipher the functional biochemical consequences of sialylation in cancer cells, a highly selective proximity labeling platform (GlycoMap) is developed, in which metabolic incorporated azidosialic acids serve as the unique reactive handle for the conjugation with DABO‐irridium via copper‐free click chemistry, making the photocatalyst only localize to sialic acids and thereby enabling the precise spatiotemporal control over labeling of sialylated proteome at the living cell surface.^[111]^ Despite these advances, it should be noted that metal photocatalysts are suffering from the difficulty to penetrate into the cell membrane. To this end, an efficient cell membrane‐permeable photocatalyst, acriflavine, was recently developed and used to profile protein‐protein interactions in the microenvironment of living cells. The HaloTag ligand functionalized with the acriflavine can be efficiently delivered into cells and localized to the HaloTag fused proteins for photocatalytic‐proximity labeling with the radical labeling reagent, MAUra, by which the proteins directly interacting with histone H2B and RNA‐binding proteins were selectively labeled within a few‐nanometer labeling radius (*CA*. 6 nm).^[112]^ In contrast to enzyme‐catalyzed proximity labeling techniques such as APEX and BioID, photocatalytic labeling strategies based on small chemical compounds such as aryl azide can provide high spatiotemporal resolution from external light irradiation and enable more focused PPI profiling in live environment.^[113]^

#### 3.2.2 Unnatural amino acids (UAAs) mediated labeling

**Figure 4:**
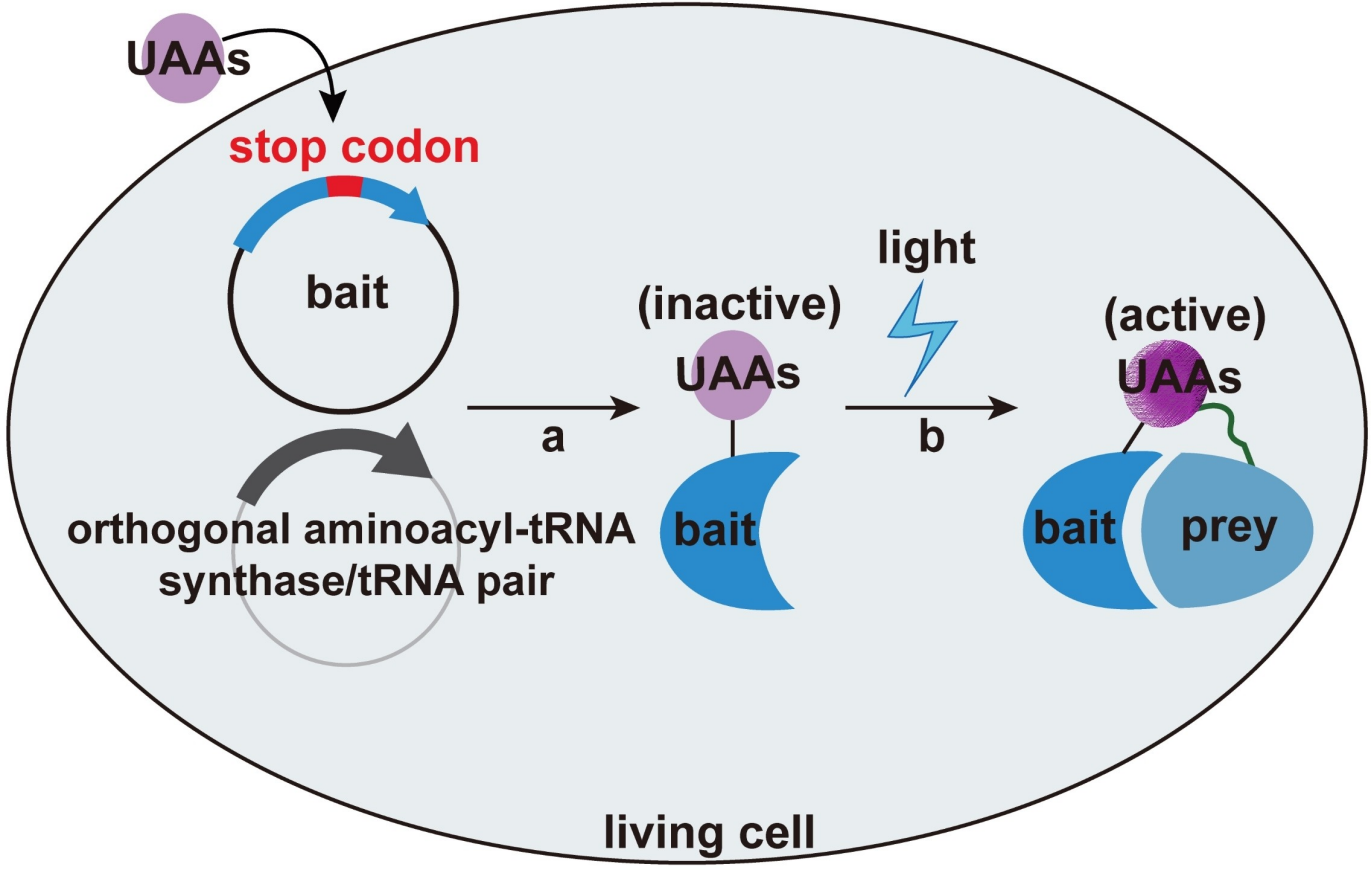
Schematic illustration of UAAs‐based methods for identifying interacting proteins. (a) The genetically encoded UAAs can be site‐specifically inserted into a bait protein by leveraging an orthogonal aminoacyl‐tRNA synthase/tRNA pair; (b) Upon UV light excitation, the photocrosslinker carried by the bait protein will be activated and can *in situ* form the covalent bond with certain residue of prey protein in the proximal environment.

**Figure 5:**
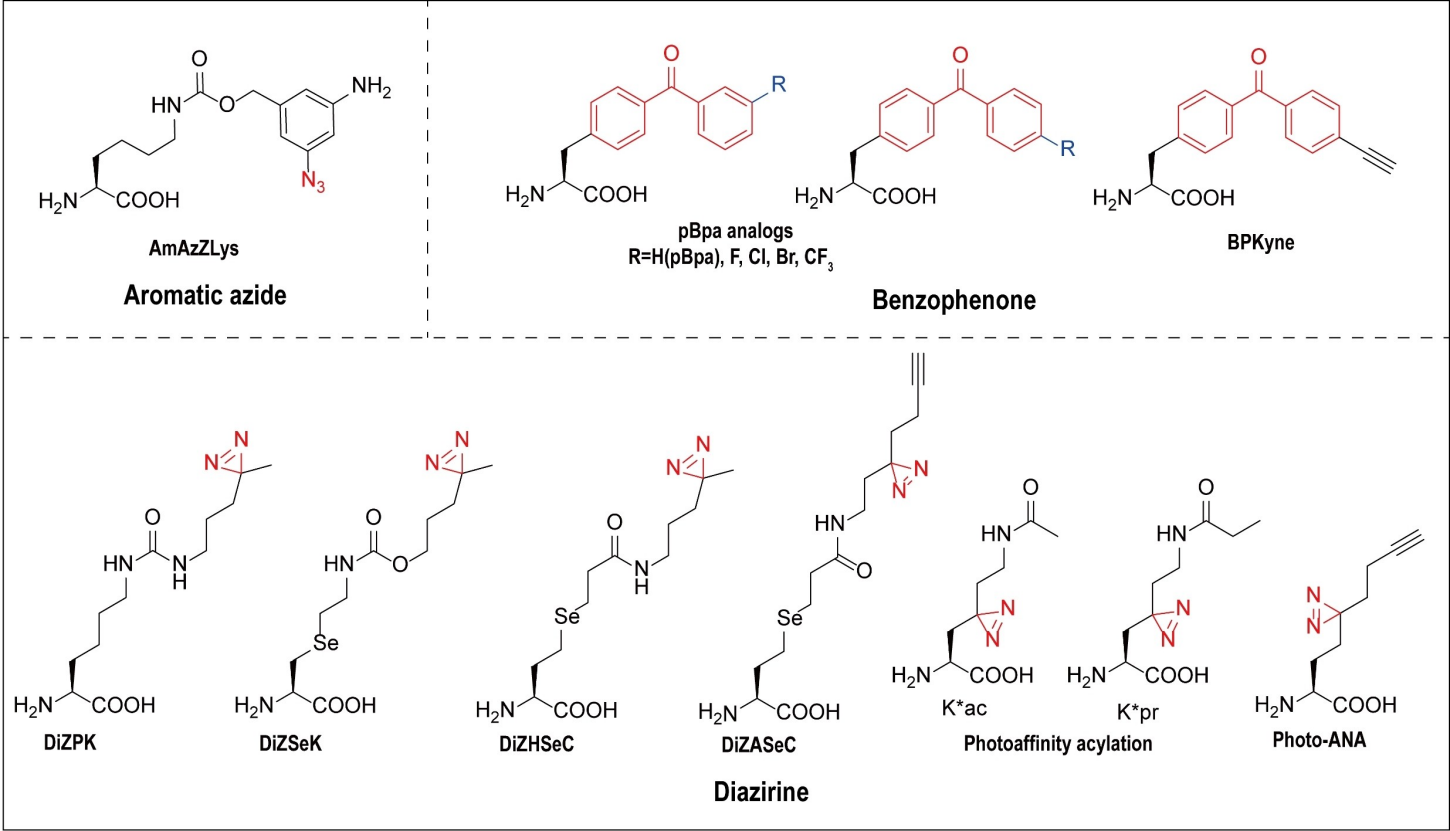
Chemical structures of some UAAs‐based photoreactive crosslinkers harboring benzoyl azide, benzophenone and diazirine moiety.

The feasibility of incorporating UAAs‐based photocrosslinkers into proteins using the genetic code expansion technology *in vivo* has been validated as another powerful tool to efficiently capture interacting proteins^[114]^ (Figure 4). The advantage of this method lies in that it can precisely control the incorporation site of UAAs on the protein, and the excited UAAs react exclusively with nearby moiety within a very limited range that depends on the length of the linker used. Moreover, different from enzymes or photocatalyst‐mediated proximity labeling approaches by which all the nearby proteins may be captured, even including a large proportion of proteins that actually have no direct interaction with the bait POI, UAAs‐based method is much more accurate and can significantly reduce false positive results. So far aromatic azide, benzophenone and diazirine‐based UAAs have emerged as three types of widely used photocrosslinkers, which can generate highly reactive intermediates upon photoactivation to react with biomolecules in proximity by forming a stable/covalent bond (Figure 5).

Aromatic azide can generate a reactive species called nitrene when exposed to light irradiation.^[115]^ For example, the bifunctional UAA (AmAzZLys) has both amino and azido groups in the benzyl moiety, which not only allows to crosslink interacting proteins under 365 nm light excitation, but also can be used for protein conjugation.^[116]^ Also, benzophenone[^117^, ^118^] reversibly forms a diradical species upon irradiation with 365 nm UV light. For instance, *p*‐benzoyl‐l‐phenylalanine (*p*Bpa) could be inserted to the adaptor protein Grb2 in Chinese hamster ovary (CHO) cells to capture with the transiently expressed epidermal growth factor (EGF) receptor in the presence of an EGF stimulus, which is a useful tool to analyze the cell signaling interactions involving Grb2.^[117]^ Due to the low reactivity of the active species, *p*Bpa‐related technology often suffers from poor photocrosslinking yields. Thus, the incorporation of halogenated *p*Bpa analogs into proteins in live yeast was developed which could increase the crosslinking yields and extend the scope of PPIs that can be profiled.^[118]^ In addition to this, one bifunctional *p*Bpa derivative 4′‐ethynyl‐*p*‐benzoyl‐l‐phenylalanine (BPKyne) containing an alkynyl moiety was also designed to achieve functionalized post‐crosslinking by using copper‐mediated azide‐alkyne Huisgen cycloaddition, which further expands the toolbox for mapping PPIs in their native environment.^[119]^ Compared to short and rigid benzophenone, reactive and diminutive diazirine^[120]^ photophore is much more frequently used due to its higher efficiency. For example, DiZPK harboring the diazirine has been used to elucidate the related proteins of chaperone protein HdeA.^[121]^ On the basis of DiZPK, a series of multifunctional UAAs probes such as DiZSeK^[122]^ and DiZHSeC^[123]^ were subsequently developed by incorporation of Se elements into them with the aim to develop a cleavable photocrosslinker to improve the identification efficiency of interacting proteins using mass spectrometry. In addition, the probe DiZASeC^[124]^ incorporating with a click handle can further reduce the background signal and allow to capture low‐abundance of target proteins. Remarkably, a powerful single‐site‐resolved multi‐omics (SiTomics) strategy has recently been developed by utilizing the bifunctional UAAs containing both lysine acylation (post‐translational modifications) and diazirine (photocrosslinker). In this study, it was found that these UAAs can be site‐specifically incorporated into the histones on the chromatin and serve as the *in situ* crosslinker to capture the interacting protein that could recognize the specific acylated lysine in native cellular chromatin, leading to the identification of GLYR1 as a distinct interacting protein in modulating H3K56cr's gene body localization.^[125]^ However, it should be noted that these activated photocatalytic intermediates are not amino acid‐specific, which may result in higher background values and complicate mass spectrometry analysis and data interpretation. Residue‐selective photo‐crosslinkers were explored to covalently lock interacting proteins to this end, such as the o‐nitrobenzyl alcohol derived lysine (o‐NBAK). Once it was incorporated into the protein of target, the corresponding aryl‐nitroso compounds via photoisomerization can be generated upon UV light activation, which are smoothly reactive towards proximal lysine to yield a stable indazolone product.^[126]^

Overall, these site‐specifically incorporated UAAs‐based strategies have proven to be useful and efficient tools to capture the potential interacting proteins with the bait POI. However, they are usually limited to map the interactomes of a single POI. Comparably, reside‐specific incorporation method is advantageous due to the capability to incorporate UAAs in a global manner. For example, a newly designed bifunctional UAA containing both diazirine and alkyne groups is recently developed and shown to be successfully incorporated into nascently produced proteosome by using the engineered methionine‐tRNA synthetase (MetRS), which provides a novel technique to profile host protein interactomes during infection at a greater proteosome scale that cannot be addressed by existing methods.^[127]^ As can be seen, UAAs‐based methods have attracted wide interest in PPIs study due to their several strengths including single‐residue resolution, and by using the new photocrosslinkers such as isoxazole,^[128]^ it can also be anticipated that more UAAs with new properties/functions will be available soon.

## 4 Conclusions and outlook

In the past decades, remarkable progresses have been made in the identification, characterization of PPIs, and their inhibitors development, which provide us great opportunities to understand the mechanisms underlying the cell signaling pathways and develop novel therapeutics for treating various diseases. Among these, the rapidly developing bio‐imaging techniques has enabled the real‐time monitoring and visualization of interaction events associated with two or more proteins of interest in living cells without disturbance to the normal functions. Also, combined with advanced and sensitive mass spectrometry technologies, various chemical cross‐linking‐mediated proximity labeling tools are particularly powerful for capturing weak/transient protein‐protein interactions *in situ*, relying on the ability to freeze protein complexes in native states by converting non‐covalent interactions into covalently‐linked bonds, which can substantially facilitate the downstream manipulation and analysis of samples to identify unknown interacting partners.

Despite these advances, most current strategies are still suffering from several drawbacks that need be addressed. First, most of them require the genetic manipulation of cells of target to express the exogenous enzymes to catalyze the labeling reaction or incorporate the orthogonal tRNA synthetase/tRNA pairs for encoding the photoreactive UAAs. Although useful and very informative, they are limited to certain types of cells that are amenable to gene transfection, but not include the primary cells and pathological samples from patients that are difficult or inert to the genetic engineering. Second, so far the available enzymes for proximal labeling have a large size and once they are fused with the protein of interest at N‐ or C‐terminus, the plausible steric hindrance from fusion proteins may affect the normal functions, for example, leading to abnormal trafficking pathway and unexpected locations in the intracellular compartments. Certain attempts to minimize such effects have been successfully explored, such as the use of a split strategy to reduce the size of fusion partners, but the addition of externally applied small compounds is always required to achieve on‐demand control of the activities of enzymes. Third, enzyme/photocatalyst‐mediated proximal labelings show their robust ability to profile the surrounding proteins in the defined microenvironments, which could yield a very informative and useful database following MS‐based analysis. But how to identify the proteins that have true/direct interaction with the protein of interest from such a large pool remains quite challenging yet, in order to elucidate the detailed functions. Towards this goal, further efforts to reduce the labeling radius may be needed to minimize the background signal arising from non‐specific labeling and increase the accuracy and efficiency of hit identification. Finally, the fact that current toolbox repertoire has made it much easier than ever before to characterize the potential interacting partners of one protein of our interest, but analyzing the multi‐interactions in complex protein networks under the same conditions in living cells remains a daunting task.

Overall, the field is still in its infancy. Undoubtedly, PPIs play a central role in regulating almost all cellular activity and the disordered PPIs will contribute to the development of various diseases, thus the ability to elucidate the complicated and enigmatic functions of PPIs in native context are being intensively pursued in the field of Chemical Biology. With such continuous endeavors made by the researchers from Biology, Chemistry and some other related disciplines, we believe that new evolving technologies with a greater degree of spatial and temporal control will be coming out soon, offering the great chances to explore PPIs non‐invasively in cells and animals, and more new discoveries and applications can be anticipated in an encouraging way.

## Conflict of interest

The authors declare no conflict of interest.

## Biographical Information

*You‐Yu Wang earned her B.S. in pharmacy from Yichun University in 2020. Currently she is studying as a Master student in the group of Prof. Xiao‐bao Bi at Zhejiang University of Technology. Her studies focus on the development of new method to profile post‐translational modification triggered protein‐protein interactome*.

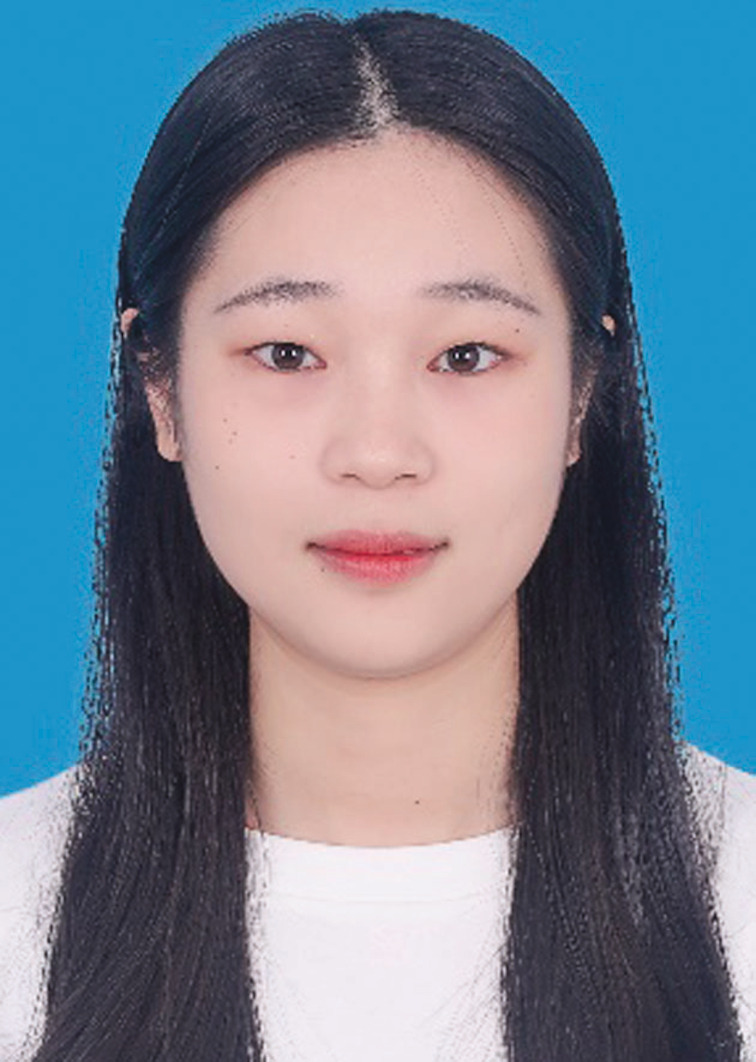

## Biographical Information

*Dr Wenyi Li obtained his doctoral degree from The University of Melbourne in 2017 working with Profs John Wade and Frances Separovic. After two postdoctoral training at FMP‐Berlin with Prof Christian Hackenberger and University of Melbourne with Profs Neil O'Brien‐Simpson and Greg Qiao, he currently works as an NHMRC Investigator EL1 and Lecturer at La Trobe University. His current research focuses on novel drug discovery by using chemical biology and peptide chemistry, especially membrane active peptides*.

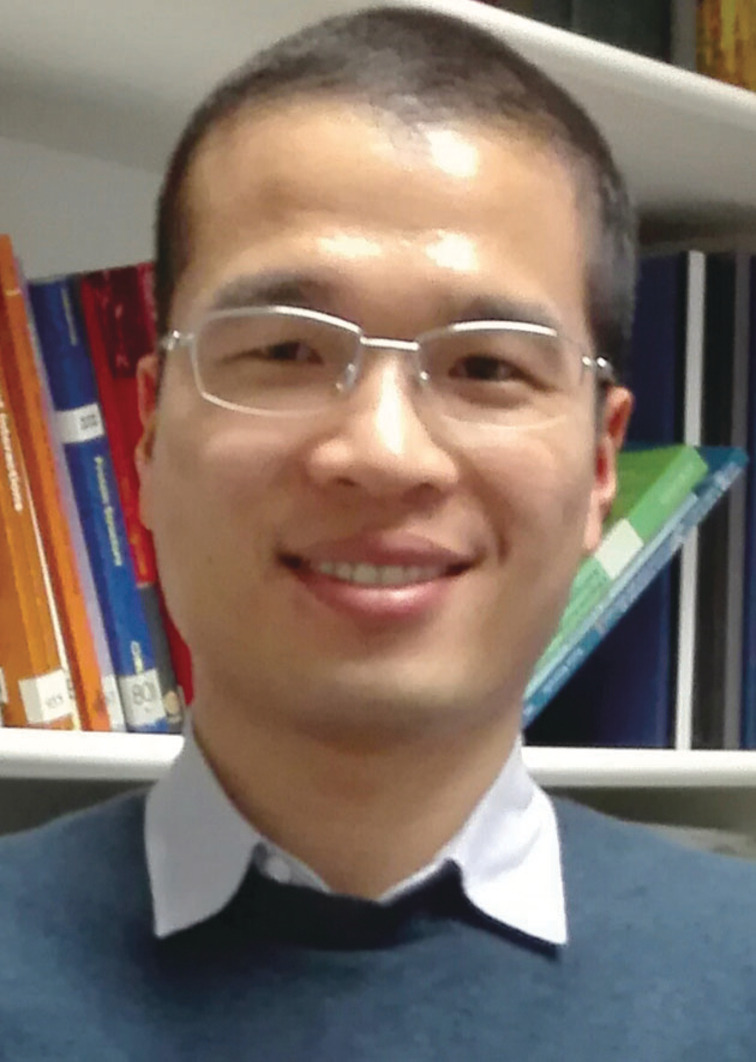

## Biographical Information

*Prof. Bang‐Ce Ye obtained his doctoral degree from East China University of Science and Technology in 1998. His research field mainly focuses on chemical biology and engineering biology*.

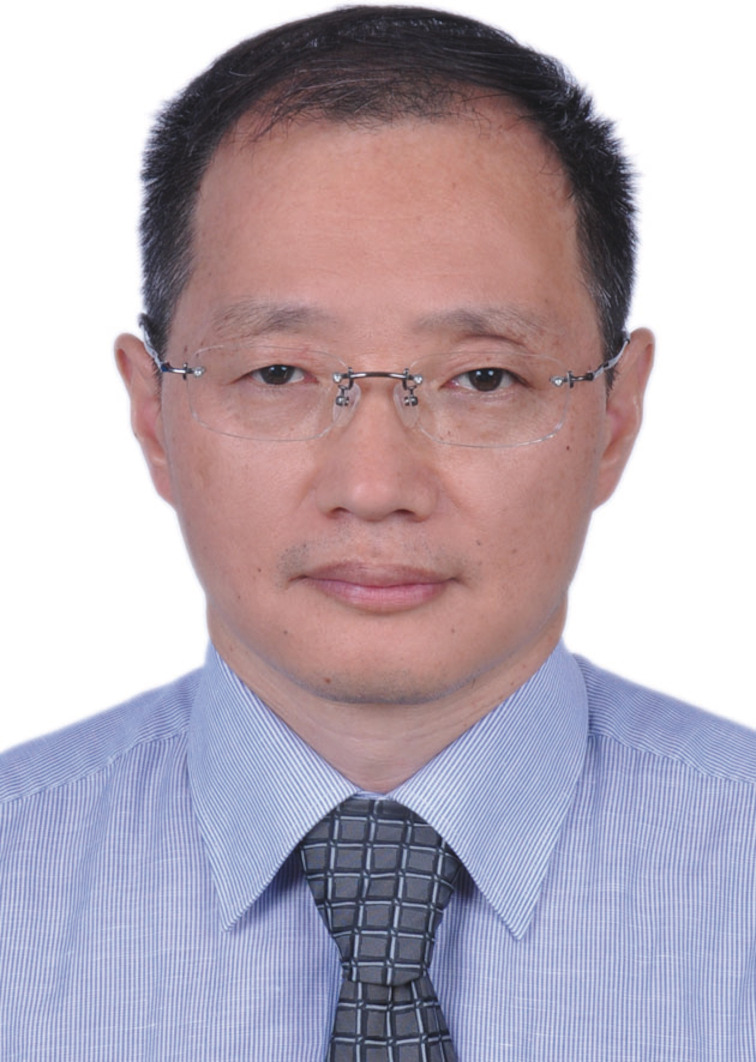

## Biographical Information

*Xiaobao Bi completed his PhD at the Nanyang Technological University in 2016 under the direction of Prof. Liu Chuan‐Fa. He started the independent work as a Professor in Zhejiang University of Technology (China) since 2021. His current research is in the fields of chemical biology and drug discovery with long‐term interest to discover peptide‐ and protein‐based therapeutics and develop novel methods for their synthesis and high‐throughput screening studies*.